# Alzheimer’s Research UK 2016 Conference report

**DOI:** 10.1186/s13195-016-0203-0

**Published:** 2016-09-15

**Authors:** Rosa M. Sancho, Carla J. Cox, Laura E. Phipps, Simon H. Ridley

**Affiliations:** Alzheimer’s Research UK, 3 Riverside, Granta Park, Cambridge, CB21 6AD UK

**Keywords:** Alzheimer’s disease, Dementia with Lewy bodies, Frontotemporal dementia, Vascular dementia

## Abstract

The annual Alzheimer’s Research UK (ARUK) Conference was hosted by the Manchester and North West Network Centre on March 8–9, 2016. In this report, we provide a summary of the research presented.

## Introduction

The Alzheimer’s Research UK (ARUK) Conference 2016 offered a diverse programme, prepared by a committee led by Nigel Hooper (University of Manchester, UK). The programme opened with an introduction from Chris and Jayne Roberts. Chris was diagnosed with mixed dementia at the age of 50 and spoke about living with the condition. Their inspiring words were followed by the latest insights from basic and clinical research.

## Frontotemporal dementia

The Conference started with an emphasis on frontotemporal dementia (FTD), an active area of research in Manchester. Jennifer Whitwell (Mayo Clinic, USA) summarised the different clinical syndromes of FTD and discussed the inclusion of primary progressive apraxia of speech, progressive supranuclear palsy and corticobasal syndrome in the FTD complex. Whitwell highlighted the neuroanatomical associations of the FTD syndromes and showed that neuroanatomical variability could be explained by different pathological associations which can be predicted by patterns of atrophy on magnetic resonance imaging (MRI). Specifically, Whitwell showed that MRI could be useful for predicting underlying pathology within patients with the behavioural variant of FTD. Offering a neuropathological perspective, Tammaryn Lashley (University College London (UCL), UK) showed changes in the expression of multiple heterogeneous nuclear ribonucleoproteins (hnRNP) between FTD-TDP-43 and FTD-FUS subtypes, highlighting the different mechanisms that could underlie these diseases. Specifically, Lashley showed that hnRNPs involved in nuclear export and not shuttled by transportin were found in pathological inclusions in FTD-FUS. Focusing on patients with C9orf72 pathology, David Mann (University of Manchester, UK) reported that, although the presence and topographic distribution of dipeptide repeats may be of diagnostic relevance, they did not relate to clinical phenotype. Conversely, the distribution and severity of TDP-43 pathology closely reflected clinical expression of disease. Mann suggested that, as the severity of TDP-43 pathology is similar in bearers and non-bearers of the C9orf72 expansion, the expansion may act as a genetic risk factor for FTD and motor neurone disease, but with clinical phenotype being driven by TDP-43 pathology. Jonathan Rohrer (UCL, UK) introduced the Genetic FTD Initiative (GENFI) 2, a five-year multi-centre study that builds on GENFI1 but with an increased number of sites across Europe and Canada. GENFI2 will aim to identify robust markers of disease onset and progression (such as cerebrospinal fluid, serum and MRI measures) as well as the optimal time to start a disease-modifying therapy. GENFI2 will also constitute a trial-ready cohort of participants for future studies.

## Disease mechanisms of dementia: inflammation

Neuroinflammation represents an important component of Alzheimer’s disease (AD) neuropathology and pathogenesis. Delphine Boche (University of Southampton, UK) highlighted the complexity of the microglial response in human AD using the Cognitive Function and Ageing Studies cohort. Her findings suggested that the microglial specific loss of Iba-1 in AD reflected the loss of microglial motility necessary to support the neurons; while microglial phagocytic proteins CD68 and MSR-A were positively associated with AD pathology and impaired cognitive function. In addition, the different microglial responses observed with either Aβ or tau in association with dementia status re-enforced that microglia have a potential role in the onset of dementia. Angela Hodges (King’s College London (KCL), UK) reported that microglia phagocytosis was impaired in AD and that this was magnified in post-mortem brains of TREM2 carriers with AD. Furthermore, Hodges showed that TREM2 carriers with AD had impaired markers of antigen presentation—HLA and Iba-1—although microglia continued to have an activated phenotype. Michael Heneka (DZNE, Germany) suggested that early and focal innate immune activation was involved at the beginning of the disease process. He focused on NLRP3 (NACHT, LRR and PYD domains-containing protein 3), an inflammasome protein whose inhibition modulated microglial functions and prevented the development of cognitive impairment in animal models of AD. Heneka described a retrospective study where pioglitazone was suggested to mediate protection from dementia, via peroxisome proliferator-activated receptor γ-mediated mechanisms. David Brough (University of Manchester, UK) proposed the use of fenamates, a class of nonsteroidal anti-inflammatory drugs, to engage targets involved in the regulation of NLRP3, in particular interleukin (IL)-1β release. Brough showed that fenamates could inhibit ASC (apoptosis associated speck-like protein containing a CARD) speck formation and inhibit NLRP3 via inhibition of the volume-regulated anion channel. In in vivo models, fenamates inhibited monosodium urate monohydrate-induced inflammation and improved the behaviour of transgenic AD mouse models, not only when administered prophylactically but also in mice displaying a phenotype. Jack Rivers-Auty (University of Manchester, UK) proposed that zinc deficiency, a common population deficiency and a prevalent deficiency in AD, exacerbated inflammation through NLRP3 and IL-1β. Rivers-Auty presented that zinc-deficient APP-PS1 mice had accelerated AD behavioural phenotype in Y-maze and Morris water maze.

## Disease mechanisms of dementia: vascular

Vascular changes can contribute to neurodegeneration in a number of different dementias. Jessica Duncombe (University of Edinburgh, UK) presented evidence to show that cortical neurovascular coupling was progressively impaired with age in wild-type mice but, surprisingly, was not further impaired in the TgSwDI mouse model of amyloid deposition. Duncombe showed that disruption of the contact between astrocytic end-feet and cerebral blood vessels, as well as increased microglial activation, could underlie this functional impairment. Focusing on patients with small vessel disease (SVD), Laura Parkes (University of Manchester, UK) presented functional imaging data from blood oxygenation level-dependent MR scans of 58 healthy participants with an age range of 18–71 years and 12 patients with SVD. The metabolic response to the Stroop task was reduced with age, suggesting that reduced neural activity/density was contributing to poorer performance. Unexpectedly, the SVD group showed increased metabolic response in comparison with age- or performance-matched control groups. This could reflect increased baseline oxygen extraction fraction in the SVD group, which could be a compensatory mechanism to maintain oxygen metabolism.

## Disease mechanisms of dementia: amyloid and prion

Understanding the underlying mechanisms of diseases causing dementia can reveal novel therapeutic targets. David Allsop (Lancaster University, UK) showed that cytotoxicity of key misfolded proteins, such as amyloid or prion protein and their fragments or mutant forms, correlated with their redox activity. Mature Aβ fibrils retained redox activity and were able to degrade H_2_O_2_ with a role for copper II (stimulatory) and/or zinc II ions (inhibitory). This resulted in the generation of highly reactive hydroxyl radicals, which could damage the protein itself or neighbouring molecules. Stephen Strittmatter (Yale, USA) presented that deletion of prion protein (PrPC) prevented the development of memory deficits in APPswe/PS1deltaE9 mice. Moreover, AD-related phenotypes could be rescued by blockade of the prion protein co-receptor, metabotropic glutamate receptor 5. Strittmatter argued that this mediated the link between PrPC and the intracellular protein mediators Homer1b/c, calcium/calmodulin-dependent protein kinase II, and protein-tyrosine kinase 2-beta. Sebastian Brandner (UCL, UK) discussed his recent findings on the iatrogenic route of transmission of prion disease by treatment of people with human growth hormone. In an autopsy study of eight individuals with iatrogenic Creutzfeldt–Jakob disease, aged 36–51 years, Brandner found moderate to severe grey matter and vascular Aβ pathology in four patients. Aβ depositions were typical of that seen in AD and cerebral amyloid angiopathy and did not co-localise with prion protein. Brandner also found Aβ deposits in the pituitary glands of patients who had AD-like pathology in the brain, suggesting that Aβ seeds were transmitted alongside the prion protein.

## Disease mechanisms of dementia: tau

Tau protein is becoming an increasingly attractive target in the search for disease-modifying treatments for AD and other tauopathies. Marie Bondulich (KCL, UK) described the characterisation of Tau35 mice, a novel model expressing human derived wild-type truncated tau at less than 10 % of the amount of endogenous mouse tau. Treated daily with phenylbutyrate for 6 weeks, these mice showed improved motor performance, spatial learning and hippocampal-dependent memory. This effect was associated with a rescue in hyperphosphorylated tau, autophagy and lysosomal markers p62, cathepsin D and acetylated tubulin as well as synapsin-1. Wendy Noble (KCL, UK) observed that the presence of activated astrocytes and phosphorylated tau in synapses correlated with dementia in AD and that, in mice, astrocytes activated by Aβ modulated tau cleavage, phosphorylation, localisation and release. Noble then presented evidence from organotypic 3xTg-AD brain slice cultures that tau relocalisation and astrocyte reactivity influenced tau release and that this could be reduced with anti-inflammatory treatment.

## Disease models: induced pluripotent stem cells

Zameel Cader (Oxford, UK) introduced the StemBANCC project, a consortium of industrial and academic partners that collects and characterises induced pluripotent stem cells (iPSCs) for several neurological diseases. StemBANCC strives to develop strong quality control metrics, as well as holding clinical information on individuals that can increase the confidence of researchers that they are working with robust, well characterised cell lines. Selina Wray (UCL, UK) is one of the scientists developing the iPSC model system for tauopathies. Wray reported that the developmental regulation of tau splicing and phosphorylation was conserved in iPSC neurons and that extended in vitro cultures are required for the expression of all tau isoforms. Splice-site mutations in *MAPT* were able to override the developmental regulation of tau splicing and led to the overproduction of 4R tau isoforms. Wray noted that long-term cultures could reveal pathological post-translational modifications. Focusing on cortical neurons generated from reprogramming and differentiation of monogenic familial AD fibroblasts, Steven Moore (ARUK Stem Cell Research Centre, University of Cambridge, UK) found that each class of mutation studied increased the production of longer Aβ peptides but did so by different mechanisms. Mutations affecting APP or an increase in its dosage led to changes in tau levels and phosphorylation, whereas several PSEN1 mutations did not. Moore also reported on progress in collaboration with the labs of Dominic Walsh (Harvard University, USA) and Michael Rowan (Trinity College Dublin, Ireland) to prospectively identify synaptotoxic peptides generated by stem cell models of dementia. To date, they have identified two distinct proteins that negatively affect synaptic function in vivo, blocking long-term potentiation induction in rodents.

## Drug discovery and development

The value of academic drug discovery complementing pharma efforts was made evident through a series of updates from across the UK. Giovanna Mallucci (University of Cambridge, UK) showed that rTg4510 mice show dysregulated PERK (RNA-activated protein kinase-like endoplasmic reticulum kinase) signalling by 6 months of age, associated with the onset of neurodegeneration. Treatment with PERK inhibitor GSK2606414 prevented further neuronal loss, reduced brain atrophy and abrogated the appearance of clinical signs. Importantly, Mallucci also showed that phenotypic screens have revealed two repurposed drugs acting on this pathway that are neuroprotective in rTg4510 and prion mice. These are safe and licensed in humans and can now be used in clinical trials of AD and dementia. Mike O’Neill (Lilly, UK) gave an overview of the amyloid and tau drug discovery programmes at Lilly and other major pharmaceutical companies. Results of several phase III trials with various amyloid therapeutic approaches will be available in the next 2–3 years. O’Neill also spoke of tau programmes being established as a priority aided by investment in cell and transgenic models of tauopathy and the development of selective positron emission tomography tracers (such as 18F-T807/AV1451) which have the potential to act as biomarkers of disease progression for future tau therapies. The Chief Scientific Officers of the ARUK Drug Discovery Alliance, John Skidmore (University of Cambridge, UK), John Davis (University of Oxford, UK) and Paul Whiting (UCL, UK), described the ongoing research opportunities available to academic researchers to translate new science into drug discovery or clinical biomarker projects. The aim of the Alliance is to generate chemical series for target validation and as leads for further development.

## Prizes for early-career dementia scientists

Rita Guerreiro (UCL, UK) was awarded the ARUK Young Investigator of the Year Award 2016 in recognition of her achievements. Guerreiro reflected on her career, which included the use of exome sequencing to identify genetic causes of rare diseases as well as risk variants for AD such as TREM2. Guerreiro highlighted the role of new technologies in allowing easier and faster genetic analysis and the fact that pleiotropic events indicate shared molecular mechanisms between neurodegenerative diseases. This year’s Jean Corsan Prize, recognising an outstanding scientific paper in neurodegeneration published by a PhD or MD/PhD student, went to Tobias Wauer (Laboratory of Molecular Biology, Cambridge, UK). During his PhD, Wauer solved the crystal structure of *Pediculus humanus* PARKIN in complex with Ser65-phosphorylated ubiquitin, revealing the molecular basis for PARKIN recruitment and activation and opening up new avenues to identify small-molecule PARKIN activators. Other prizes awarded to conference attendees were: the Dick Bell Prize for Communication to Jessica Duncombe, the David Dawbarn Poster Prize to Patricia Vazquez Rodriguez (University of Cambridge, UK) and the Manchester and North West ARUK Network Centre Poster Prize to Elijah Mak (University of Cambridge, UK).

## Abbreviations

AD: Alzheimer’s disease
ARUK: Alzheimer’s Research UK
FTD: Frontotemporal dementia
GENFI: Genetic FTD Initiative
hnRNP: Heterogeneous nuclear ribonucleoprotein
IL: Interleukin
iPSC: Induced pluripotent stem cell
KCL: King’s College London
MRI: Magnetic resonance imaging
PrPC: Prion protein
SVD: Small vessel disease
UCL: University College London

